# Modified Whale Optimization Algorithm based ANN: a novel predictive model for RO desalination plant

**DOI:** 10.1038/s41598-023-30099-9

**Published:** 2023-02-18

**Authors:** Rajesh Mahadeva, Mahendra Kumar, Vinay Gupta, Gaurav Manik, Shashikant P. Patole

**Affiliations:** 1 Department of Physics, Khalifa University of Science and Technology, 127788 Abu Dhabi, United Arab Emirates; 2 Department of Instrumentation and Control Engineering, Dr. B R Ambedkar National Institute of Technology, Jalandhar, Punjab 144011 India; 3 Department of Polymer and Process Engineering, Indian Institute of Technology, Roorkee, Uttarakhand 247667 India

**Keywords:** Environmental sciences, Engineering

## Abstract

In recent decades, nature-inspired optimization methods have played a critical role in helping industrial plant designers to find superior solutions for process parameters. According to the literature, such methods are simple, quick, and indispensable for saving time, money, and energy. In this regard, the Modified Whale Optimization Algorithm (MWOA) hybridized with Artificial Neural Networks (ANN) has been employed in the Reverse Osmosis (RO) desalination plant performance to estimate the permeate flux (0.118‒2.656 L/h m^2^). The plant’s datasets have been collected from the literature and include four input parameters: feed flow rate (400‒600 L/h), evaporator inlet temperature (60‒80 °C), feed salt concentration (35‒140 g/L) and condenser inlet temperature (20‒30 °C). For this purpose, ten predictive models (MWOA-ANN Model-1 to Model-10) have been proposed, which are capable of predicting more accurate permeate flux (L/h m^2^) than the existing models (Response Surface Methodology (RSM), ANN and hybrid WOA-ANN models) with minimum errors. Simulation results suggest that the MWOA algorithm demonstrates a stronger optimization capability of finding the correct weights and biases so as to enable superior ANN based modeling without limitation of overfitting. Ten MWOA-ANN models (Model-1 to Model-10) have been proposed to investigate the plant’s performance. Model-6 with a single hidden layer (*H* = 1), eleven hidden layer nodes (*n* = 11) and the thirteen search agents (*SA* = 13) produced most outstanding regression results (R^2^ = 99.1%) with minimal errors (MSE = 0.005). The residual errors for Model-6 are also found to be within limits (span of − 0.1 to 0.2). Finally, the findings show that the screened MWOA-ANN models are promising for identifying the best process parameters in order to assist industrial plant designers.

## Introduction

This section has been separated into three parts: the first part describes the background of ANN and WOA, while the second part details the literature review. The third part explains the major objectives, contributions, and research outline.

### Background

The human ambition to do tasks more quickly, easily and inexpensively has led to the increasing development of efficient operations worldwide^1,2^. In the same way, the process plant industry is changing to a culture where decisions are being made based on data analysis and experimental outcomes^3,4^. In this regard, the plant’s experimental datasets have been collected and evaluated to gather new insights, which aids in decision-making for plant designers to save processing time, operational cost, and energy^1,5,6^.

In recent decades, process plant industries have become considerably more dynamic and have turned to advanced analytics, optimization algorithms, and machine learning tools to provide predictive and prescriptive solutions to improve their performance^3,5–11^. These algorithms and tools are simple, adaptable, and efficient for analyzing datasets of small as well as large-scale plants. Some commonly used intelligent algorithms and tools being used lately include Artificial Neural Networks (ANN)^12–15^, Artificial Bee Colony (ABC)^16,17^, Cat Swarm Optimization (CSO)^18,19^, Particle Swarm Optimization (PSO)^20–22^, Firefly Algorithm (FA)^23^, Bat Algorithm (BA)^23,24^, Whale Optimization Algorithm (WOA)^17,25–27^, Grey Wolf Optimizer (GWO)^17,25,28–30^ Butterfly Optimization Algorithm (BOA)^31^, Ant Lion Optimizer (ALO)^17^, Support Vector Machine (SVM)^18,32,33^, Response Surface Methodology (RSM)^34,35^, Non-Dominated Sorting Genetic Algorithm (NSGA)^36^ and their hybrid.

#### Problems with ANN

ANN, in general, follows the backpropagation (BP) training algorithm while it finds an optimum set of node connection weights and biases to reduce the error. An accurate prediction of weights and biases is very important to ensure high model performance. The BP approach employs a gradient descent algorithm and necessitates a large number of iterations^37^. Literature suggests that one of the biggest challenges in using the gradient descent technique is its trapping in the local optima. This is entirely tied to the initial values of weight considered^37^, which affects the final accuracy of the models. Therefore, researchers have found alternative solutions such as GA, PSO, GWO, and WOA to minimize these issues^1,6^.

#### Why hybridization?

Hybridization is now the most widely used technology for analyzing a plant’s performance precisely as it combines two algorithms and tools into one and allows them to work synergistically^1,2,6^. Literature suggests various hybrid models, such as GA-ANN^25,38^, PSO-ANN^21^, ABC-ANN^16^, SVM-ANN^33^, PSO-SVM^32^, WOA-ANN^39^, and others, have focused on system model effectiveness in the investigation of the various fields of engineering and in assessing plant’s performance. Among them, ANN with hybrid models is the most extensively utilized technology to investigate plant datasets correctly^5,6^.

#### Why modified WOA in ANN?

With the motivation of literature^26,39^, we have developed hybridized models using modified WOA (MWOA) with ANN to model and analyze the performance of reverse osmosis (RO) desalination plants. The models are then simulated to assess the capability of such hybridization so as to find the optimum biases and weights used in algorithms to increase the ANN model’s accuracy and precision. More specifically, this paper explores the possibility of using the MWOA algorithm in the ANN model to overcome the limitations of BP training algorithms for improving model performances and, thereby, enabling better modeling of desalination processes and realization or prediction of its performance. The datasets used previously by Gil et al.^35^ have been utilized here as well so as to make a comparison with results published by them. It is observed from the simulation that modified WOA serves as a superior optimization for ANN in this investigation compared to BP-assisted ANN used earlier^35^ and simple WOA algorithms.

### Literature review

As stated in the background subsection, many researchers have been using ANN and their hybrid models to examine plant performance. Some of them are particularly relevant to desalination plants and ANN modeling, which we discuss further in this section to help better understand the significance of such models. Lee et al.^40^ developed an ANN model to predict the permeate total dissolved solids (TDS) (354.2 to 745.7 ppm) and permeate flow rate (454.0 to 470.2 m^3^/h) of the seawater RO desalination plant. They have investigated a one-year operation dataset of the Fujairah seawater RO desalination plant, United Arab Emirates (UAE)^40^. The entire dataset was divided into three parts for modeling investigations: 60% for training, 20% for testing, and 20% for validation. They predicted permeate TDS (regression coefficient, R^2^ = 96%) and permeate flow rate (R^2^ = 75%) for the testing stage. Further, Aish et al.^12^ proposed a multilayer perceptron (MLP) neural network and radial basis function (RBF) neural network to predict TDS concentrations (training 10 to 430 mg/L and testing 11.80 to 340 mg/L) and permeate flow rate (training 9.5 to 17 bars and testing 10 to 15.5 bars) of RO desalination plant, Gaza Strip, Palestine^12^. The data was collected for over six months (March to September 2013) and divided into two parts, 70% for training and 30% for testing. They have reported the best-predicted TDS concentrations with minimum error (Mean Squared Error, MSE = 0.023) for testing of the MLP model. In addition, they have also reported the best-predicted permeate flow rate with minimum error (MSE = 12.645) for testing the RBF model.

Likewise, Cabrera et al.^13^ developed models to assess the optimal operating pressure (bars) and feed flow rate (m^3^/h) of an RO desalination plant, Gran Canaria, Spain, using the ANN model. While modeling, they utilized 505 sets of data and reported a good agreement between the predicted and experimental outcomes with minimum errors (0.026 m^3^/h) for feed flow rate and (0.252 bars) for operating pressure. They have also reported using a large number of 38 and 56 nodes in the first hidden layer and 4 and 9 nodes in the second hidden layer as most suitable for the proposed ANN modeling. Recently, Panahi et al.^41^ proposed a hybrid ALO-ANN model to predict clean water production in seawater greenhouses in arid lands. They reported that the ALO-ANN model outperformed the ANN, BA-ANN, and PSO-ANN in the testing phase, with RMSE % values of 39, 18, and 33%, respectively, lower than that of the ANN, BA-ANN, and PSO-ANN models.

Recent studies of WOA and their variants motivate researchers to work in this field, such as Fu et al.^42^ utilized nicely hybrid long short‑term memory with WOA and variational modes to estimate monthly evapotranspiration. Ding et al.^43^ proposed three improved versions of the WOA to enhance the exploration abilities, also employed to improve population diversity. Similarly, Ju et al.^44^ suggested a hybrid strategy of WOA based on nonlinear convergence factor, chaos initialization, and mutation concepts. Further, Chakraborty et al. proposed various artificial intelligence models using WOA and their variants for numerous applications, such as for COVID-19 X-ray image segmentation^45^, global optimization^46,47^, numerical optimization^48^, and other applications^49–52^.

Literature reveals that the accurate achievement of the model’s targets depends on the specific selection of the algorithms and the modeling parameters. Literature also suggests that nature-inspired algorithms have excellent search capabilities to achieve global optima. In addition, these algorithms are able to adjust themselves as per the objective functions. But some algorithms and models, such as BP-ANN, have limitations in finding the global minima. In this context, this study focuses highly on WOA algorithms because of their uniqueness and capability to find optimum weights and biases in the global optima. Therefore, this investigation employs a Modified WOA (MWOA) algorithm to achieve the global optima and support ANN for an accurate outcome with minimum errors. For this, we have employed reverse osmosis (RO) desalination plant datasets for investigating and validating the results with the existing models.

The primary focus of this research is to investigate the use of artificial intelligence technologies in the fields of desalination and water treatment. However, many researchers have worked in this area and produced several models for improving plant performance. Yet, to the best of our knowledge and over literature review, the MGWO-ANN technique is being proposed and applied to the modeling of the RO desalination plant for the first time.

### Major objectives, contributions, and outline

According to WHO and UNICEF reports (2017)^53^, By 2025, ‘half of the world’s population may live in water-scarce places’. Therefore, it is imperative for researchers to accelerate research in the improved desalination field to ensure a sustainable life for humans, animals, and plants. We intend to promote this by utilizing the modified WOA algorithm in ANN to appropriately model such systems and improve process parameter prediction of desalination plants. According to the literature findings and our best knowledge, the hybrid MWOA-ANN models have been employed herewith for the first time to predict the RO desalination plant permeate flux (0.118‒2.656 L/h m^2^).

The remaining part of the paper is organized as follows: Section “Datasets and methodology” defines the datasets and methodology, whereas Section "Results and Discussion" describes the results and discussion. Finally, in Section “Conclusion”, the conclusion of this work is presented.

## Datasets and methodology

This section has been separated into two parts: the first part describes the datasets used in this investigation, while the second part details the proposed methodology. The second part explains the concepts of ANN, MWOA, and the hybrid MWOA-ANN models employed herewith for a better understanding of the model developed by the reader.

### Dataset details

In this research investigation, the desalination plant’s experimental datasets from the previous work by Gil et al.^35^ have been used for the proposed modeling. The plant module referred by them was designed by the Fraunhofer Institute for Solar Energy Systems that uses a W. L. Gore Associates commercial membrane [Permeate Gap Membrane Distillation (PGMD)] with an active Polytetrafluoroethylene (PTFE) layer^35^. Four input parameters: salt concentration, flow rate, evaporator, and condenser intake temperatures were used, while permeate flux was the output parameter of the model. The details of the ranges of parameters are presented in Table 1^35^.

**Table 1:** Parameters involved in the proposed modeling of RO desalination plants^35^.

A	Parameters (feed/input)	Parameters value with range
(i)	Condenser inlet temp. (*T*_cond_)	20–30 °C
(ii)	Evaporator inlet temp. (*T*_evap_)	60–80 °C
(iii)	Feed flow rate (*F*)	400–600 L/h
(iv)	Feed salt concentration (*S*)	35–140 g/L

B	Parameter (permeate/output)	
(i)	Permeate flux, (*P*_flux_)	0.118–2.656 L/h m^2^

### Proposed methodology

#### Artificial neural network (ANN) architecture

**Figure 1:**
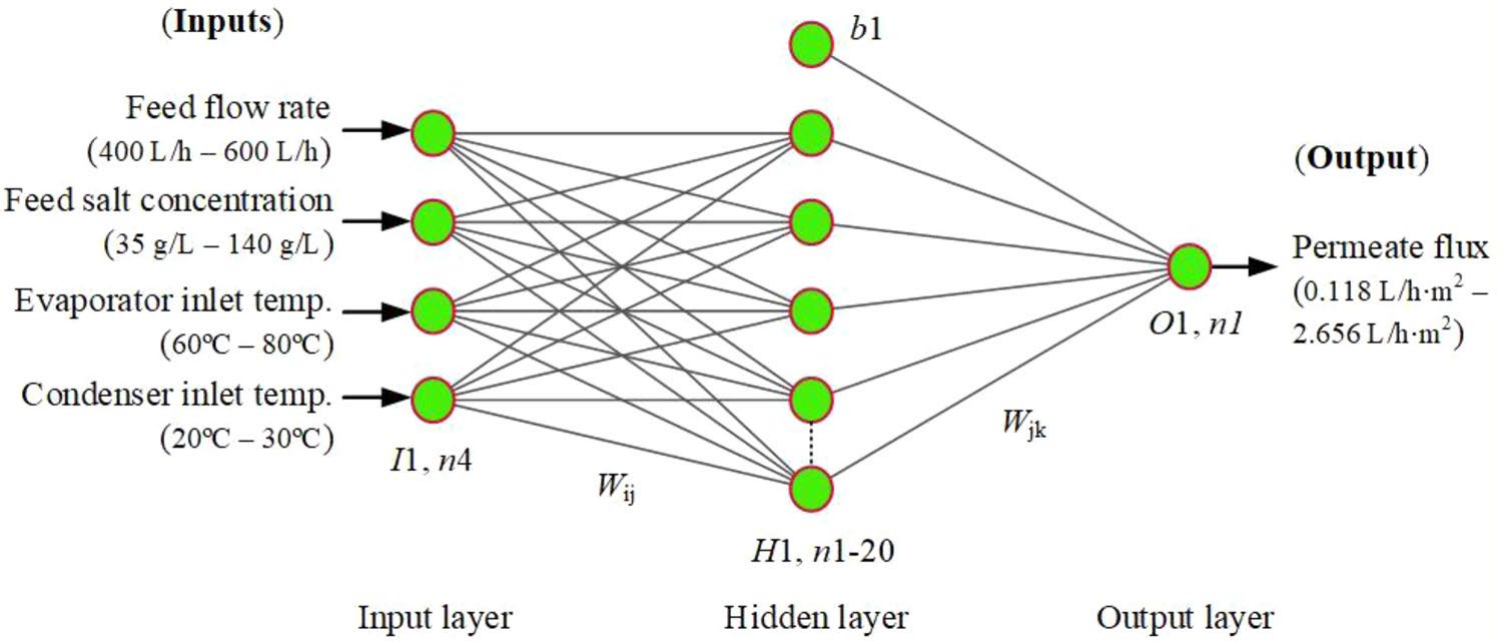
Illustrates the basic ANN architecture {(*I*1, *n*4): (*H*1, *n*1–20): (*O*1, *n*1)}.* b*1 represents single bias, *W*_ij_ represents weights between the input and hidden layers, and *W*_jk_ represents weights between the hidden and output layers.

ANN is the elementary model of this research, which has been improved in this investigation through its hybridization with an advanced optimization process. It is based on the activity of biological neurons in human brains, and the concept of neural network learning was first proposed by McCulloch and Pitts^54^. It showed a strong capacity to anticipate various engineering applications’ performance and effectively handle complex, linear, and nonlinear tasks. In the literature, ANN architectures such as generalized regression neural network (GRNN), radial basis function (RBF), and multilayer perceptron (MLP) are suggested, with MLP being the most prevalent and frequently used in numerous applications^1^. In general, ANN employs three layers: (input, hidden, and output), and follows the backpropagation (BP) learning technique with a Levenberg–Marquardt (LM) training algorithm^5^. The models map the relationship between inputs and targets^5,6^. We have proposed an architecture of this type {(*I*1, *n*4): (*H*1, *n*1–20): (*O*1, *n*1)} as illustrated in Fig. 1. Here, (*I*1, *n*4) represents a single input layer with four nodes, (*H*1, *n*1–20) represents a single hidden layer with 1 to 20 nodes, and (*O*1, *n*1) represents a single output layer with one node.

#### Modified Whale Optimization Algorithm (MWOA)

**Figure 2:**
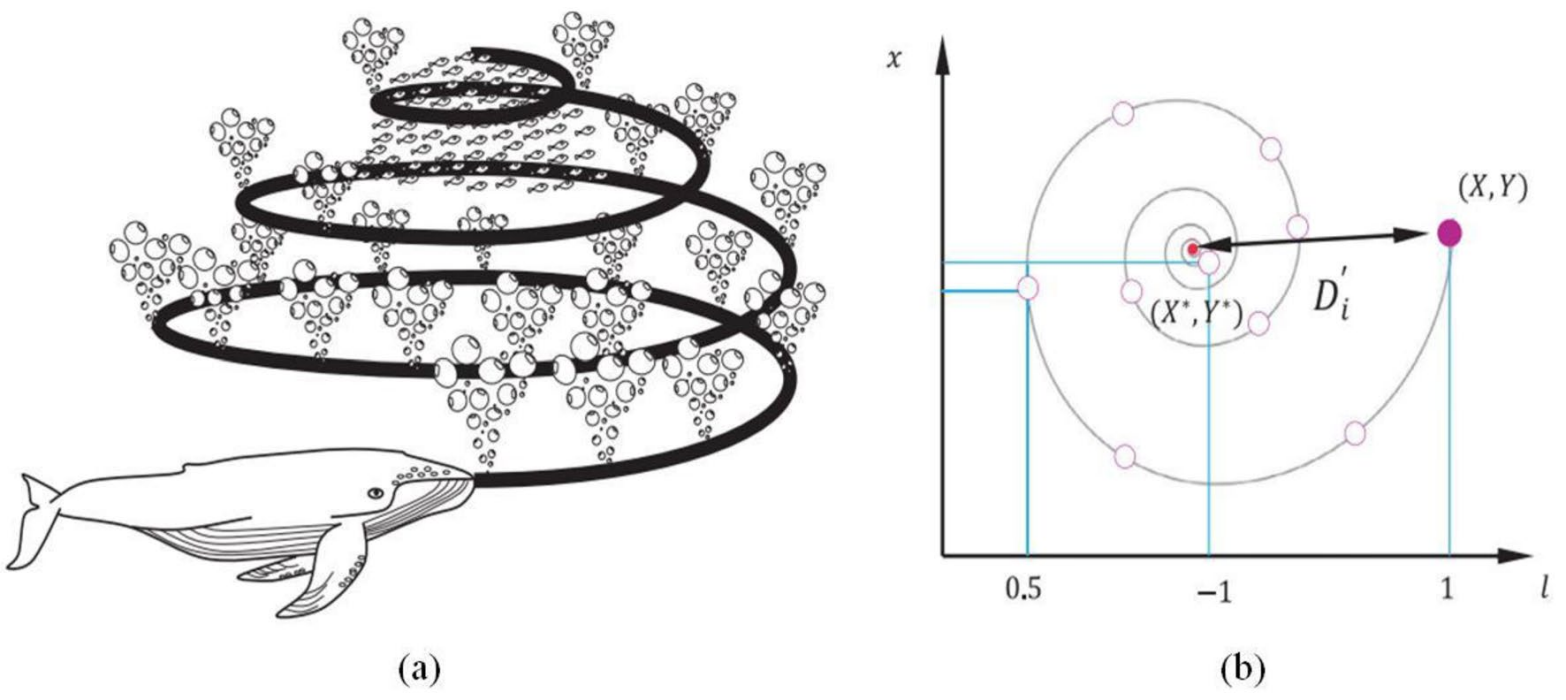
(**a**) Bubble-net feeding behavior of humpback whales (**b**) Spiral updating position. Photo: Courtesy Mirjalili and Lewis^27^.

Whales are the world’s largest mammals and the most beautiful creatures in nature. Whales have spindle cells in their brains that are similar to ‘human spindle cells’ and are responsible for emotions, judgment, and social behaviors, according to Hof and Gucht^55^. They have fantastic behavior in that they can live alone or in groups. In addition, the fascinating aspect of ‘humpback whales’ is their unique hunting technique, known as bubble-net feeding^27^. This hunting skill focuses on forming various bubbles along a ‘9’ shaped path or circle, as displayed in Fig. 2, which helps the humpback whales finally catch the smaller fishes near the surface of the water^27^.

##### The mathematical formulation of MWOA

The mathematical formulation of MWOA involves three steps, namely, Step 1: Encircling prey, Step 2: Bubble-net attacking method (Exploitation Phase), and Step 3: Search for prey (Exploration Phase)^27,56^.

**Figure 3:**
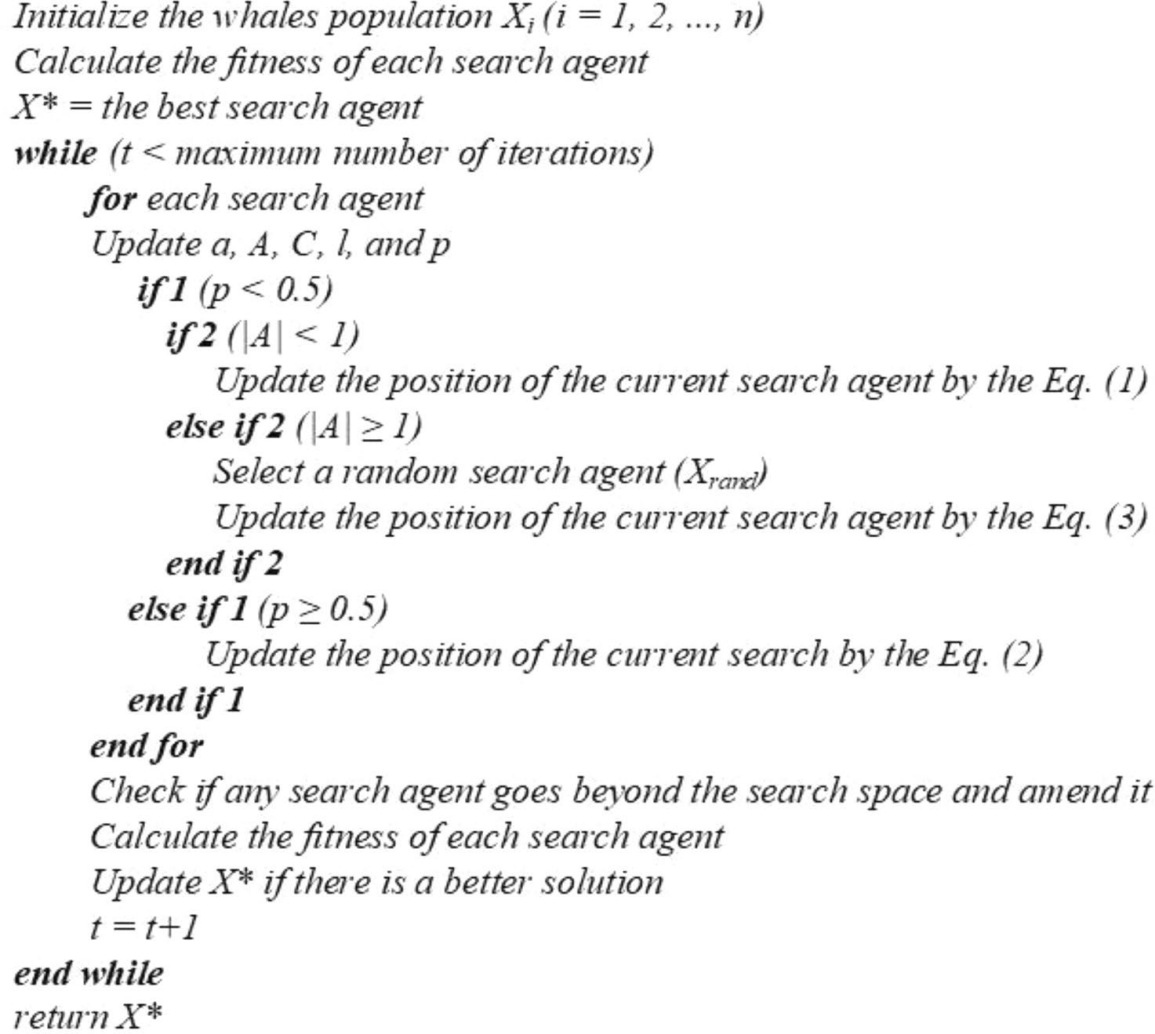
The MWOA algorithm's pseudo-code. Photo: Courtesy Mirjalili and Lewis^27,56^.

Step 1.: Encircling prey^27,56^:
  Whales first locate their prey and then start to encircle them. They try to estimate the best candidate solution, also known as the best search operator (BSO), and then update their positions accordingly to match the BSO. The following equations mathematically represent this behavior:
  1 $$ \vec{D} = \left| {\vec{C} \cdot \vec{X}^{*} (t) - \vec{X}(t)} \right|;\;\;\;\vec{X}(t + 1) = \vec{X}^{*} (t) - \vec{A} \cdot \vec{D} $$
  where, $\vec{D}$ = displacement in the position of the prey; $\vec{X}^{*}$ = position vector of the best solution obtained so far; $\vec{X}$ = position vectors; _*t*_ = current iteration; $\vec{A}$ and $\vec{C}$ = coefficient vectors; $\vec{A} = 2\vec{a} \cdot \vec{r}_{1} - \vec{a}$ and $\vec{C} = 2 \cdot \vec{r}_{2}$; $\vec{r}_{1}$ and $\vec{r}_{2}$ random vectors in [0, 1]; $\vec{a} = 2\left( {1 - \frac{{t^{2.5} }}{{t_{m}^{2.5} }}} \right)$; and _*tm*_ = maximum iterations.
Step 2.: Bubble-net attacking method (Exploitation Phase)^27,56^:
  As stated earlier, whales swim in a ‘9’-shaped path around the prey in the shrinking circle, as illustrated in Fig. 2. This technique has been found to form various bubbles in water along a circle. This is simulated by choosing a shrinking encircling scheme (Fig. 2b) with a 50% chance during iterations. Thus, the following equations mathematically represent this bubble-net attacking behavior as:
  2 $$\overrightarrow{X}\left(t+1\right)=\left\{\begin{array}{ll} \vec{X}^{*} \left( t \right) - \vec{A} \cdot \vec{D} & \quad  if \; p<0.5\\ \vec{D}^{{\prime}}  \cdot e^{{bl}}  \cdot \cos \left( {2\pi l} \right) + \vec{X}^{*} \left( t \right) & \quad if \; p\ge 0.5\end{array}\right.$$
  where, *l* is the random number in [− 1, 1], *p* is the arbitrary number in [0, 1]; and *b* is the constant (for identifying logarithmic spiral shape).
Step 3.: Search for prey (Exploration Phase)^27,56^:
  In the exploration stage, rather than the exploitation stage, the position of the search operator is updated using a randomly chosen search operator ($\vec{X}_{rand}^{*}$). This strategy will emphasize exploration while also allowing MWOA to complete a global search. For the exploratory phase, the following equation is used:
  3 $$ \vec{D} = \left| {\vec{C} \cdot \vec{X}_{rand}^{*} (t) - \vec{X}(t)} \right|;\;\;\;\vec{X}(t + 1) = \vec{X}_{rand}^{*} (t) - \vec{A} \cdot \vec{D} $$
  where, $\vec{X}_{rand}^{*}$ is the position vector (random) selected from the current population. In addition, the MWOA algorithm’s pseudo-code is shown in Fig. 3^27,56^. MWOA can be called a global optimizer from a theoretic point of view because it contains collective exploitation and exploration capability.

#### Proposed MWOA-ANN models

**Figure 4:**
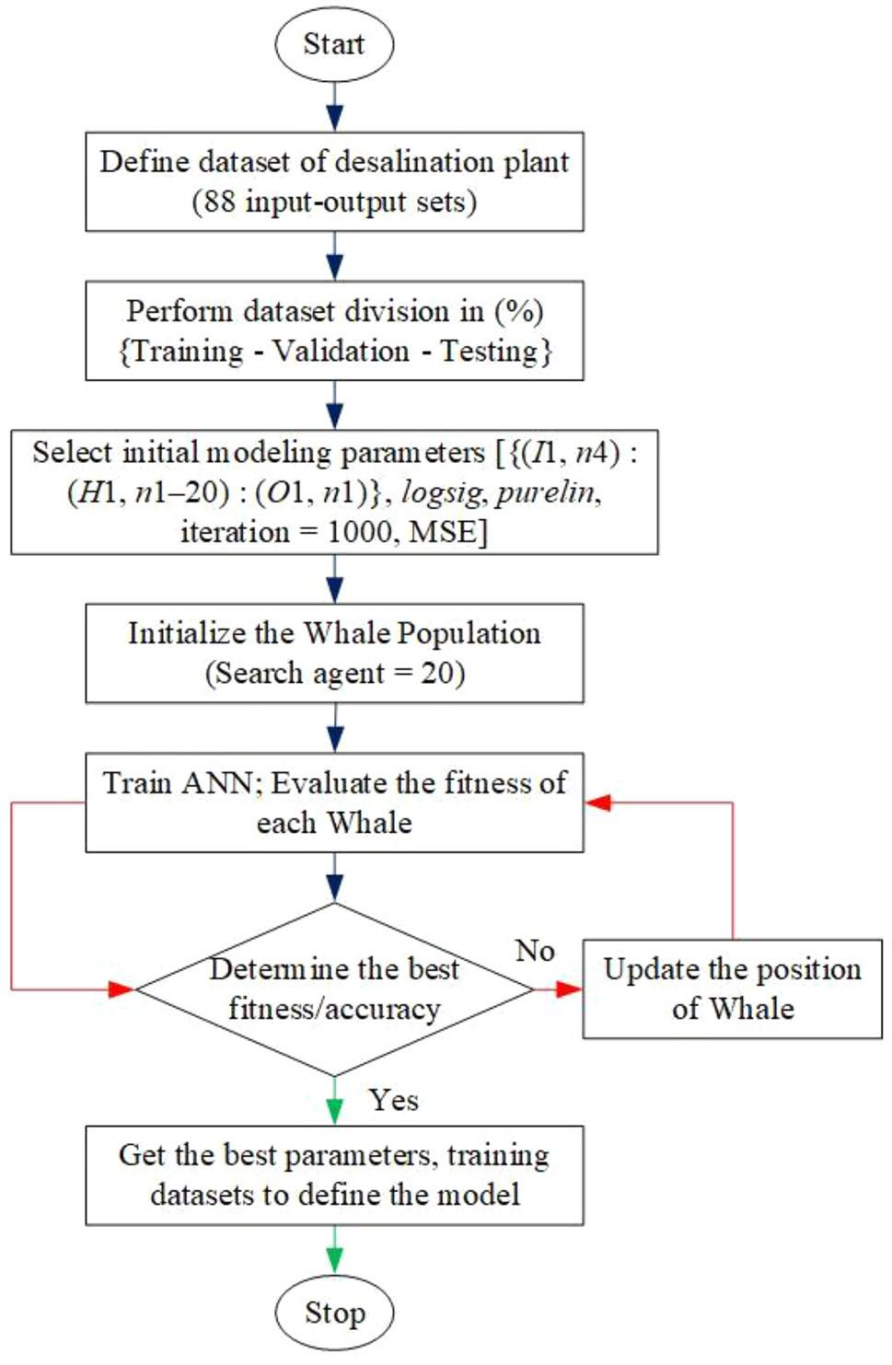
Flow diagram of the proposed (MWOA-ANN) model.

According to the literature, several hybrid models accurately predict the performance of diverse domains. This study used the MWOA technique to train the ANN model. For this, we have suggested ten hybridized models with ANN (MWOA-ANN Model-1 to Model-10) to estimate the RO desalination plant’s performance. Thus, the vital objective of this study is to minimize the error (least MSE). Hence, the error (MSE) is defined as the following^21,22,40^:

4 $$ {\text{MSE }} = \frac{1}{2N}\sum\limits_{p = 1}^{N} {\sum\limits_{k = 1}^{M} {\left( {y_{k}^{p} - \hat{y}_{k}^{p} } \right)^{2} } } $$

where, $\hat{y}_{k}^{p}$ predicted output of the neural network, $y_{k}^{p}$ real output; *M* no. of output nodes and* N* no. of patterns. The complete flow diagram of the suggested model (MWOA-ANN) is displayed in Fig. 4. Primarily, collect the RO desalination plant data and define the data sets. In this work, we have collected datasets from previous work by Gil et al.^35^. Then, according to the model’s computational requirements, we arrange the data and execute dataset division (%) into training, validation, and testing. For simulating the model, the appropriate initial modeling parameters are selected. Then, the whale population or search agent (*SA*) is initialized and each whale’s fitness is evaluated. Further, determine the best fitness; if it meets the desired requirement or criteria, then record and stop; otherwise, update the whale’s position and re-evaluate the fitness until the desired fitness is achieved.

## Results and discussion

This section has been divided into three parts to comprehend the research findings better: “Optimization”, “Best-optimized models”, and “Best-of-best optimized model and their novelty”.

### Optimization

Literature suggests a model’s accuracy is dependent on a perfect design and a systematic approach of the model. The right choice of modeling parameters and appropriate dataset divisions makes the model design perfect. In addition, the best model is a step-by-step systematic approach carried out in a proper manner. As a result, we have employed both principles to improve our model and choose the finest models in this section. For the best selection of the model, we have optimized three important modeling parameters [*n*, *SA*, and dataset division (%)] step-by-step in a systematic manner and achieved various fruitful outcomes. The best model selection criteria are better outcomes than the existing models (RSM and basic ANN structure)^35^.

#### The number of hidden layer nodes (*n*) optimization

**Figure 5:**
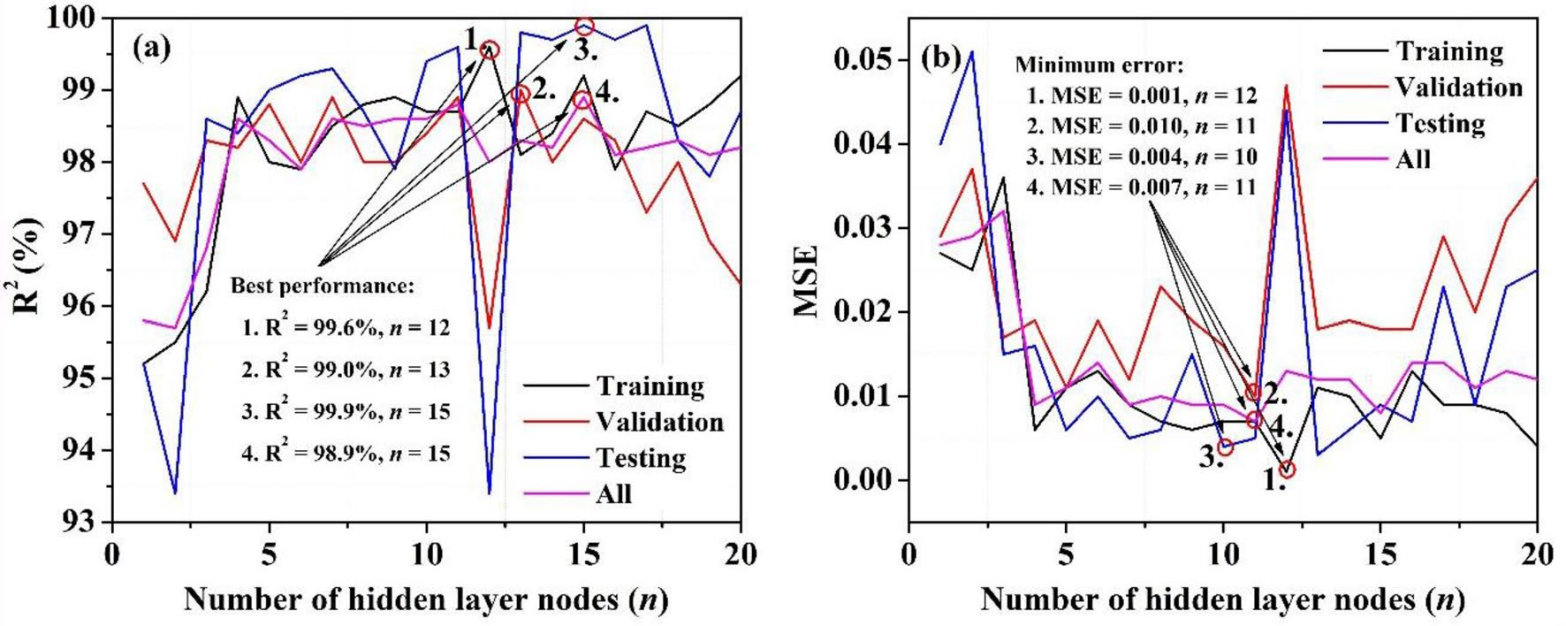
Optimization of hidden layer nodes (*n*) for different stages (training, validation, testing, and all) for: (**a**) Regression coefficients (R^2^), (**b**) MSE.

The number of hidden layer nodes (*n*) plays an essential role in optimizing the model. In order to do this, we varied the hidden layer nodes one by one (*n* = 1 to 20), and the results obtained are presented in Table 2. For easier comprehension and evaluation, the results are also shown graphically in Fig. 5. We observed that though models with *n* = 12 and 13 demonstrate the best performance for training and validation individually, the model with *n* = 15 yielded the best performance for testing and all datasets. Finally, we screened two models (with *n* = 11 and 15), which achieved our selection criteria for best simulation results (highest R^2^ = 98.8, 98.9% and lowest MSE = 0.007, 0.008) and recorded them as favorable models.

**Table 2:** Permeate flux prediction at various stages using the proposed MWOA-ANN model.

Number of hidden layer nodes (*n*)	Results {*P*_flux_ (L/h m^2^)}							
	Training		Validation		Testing		All	
	R^2^ (%)	MSE	R^2^ (%)	MSE	R^2^ (%)	MSE	R^2^ (%)	MSE
1	95.2	0.027	97.7	0.029	95.2	0.040	95.8	0.028
2	95.5	0.025	96.9	0.037	93.4	0.051	95.7	0.029
3	96.2	0.036	98.3	0.017	98.6	0.015	96.8	0.032
4	98.9	0.006	98.2	0.019	98.4	0.016	98.6	0.009
5	98.0	0.011	98.8	0.011	99.0	0.006	98.3	0.011
6	97.9	0.013	98.0	0.019	99.2	0.010	97.9	0.014
7	98.5	0.009	98.9	0.012	99.3	0.005	98.6	0.009
8	98.8	0.007	98.0	0.023	98.7	0.006	98.5	0.010
9	98.9	0.006	98.0	0.019	97.9	0.015	98.6	0.009
10	98.7	0.007	98.4	0.016	99.4	0.004	98.6	0.009
11*	98.7	0.007	98.9	0.010	99.6	0.005	98.8	0.007
12	99.6	0.001	95.7	0.047	93.4	0.044	98.0	0.013
13	98.1	0.011	99.0	0.018	99.8	0.003	98.3	0.012
14	98.4	0.010	98.0	0.019	99.7	0.006	98.2	0.012
15*	99.2	0.005	98.6	0.018	99.9	0.009	98.9	0.008
16	97.9	0.013	98.3	0.018	99.7	0.007	98.1	0.014
17	98.7	0.009	97.3	0.029	99.9	0.023	98.2	0.014
18	98.5	0.009	98.0	0.020	98.3	0.009	98.3	0.011
19	98.8	0.008	96.9	0.031	97.8	0.023	98.1	0.013
20	99.2	0.004	96.3	0.036	98.7	0.025	98.2	0.012

The number of hidden layer nodes was selected as the optimization modeling parameter.

*Significant predicted values for all datasets.

#### The number of whales population/search agents (SA) optimization

**Figure 6:**
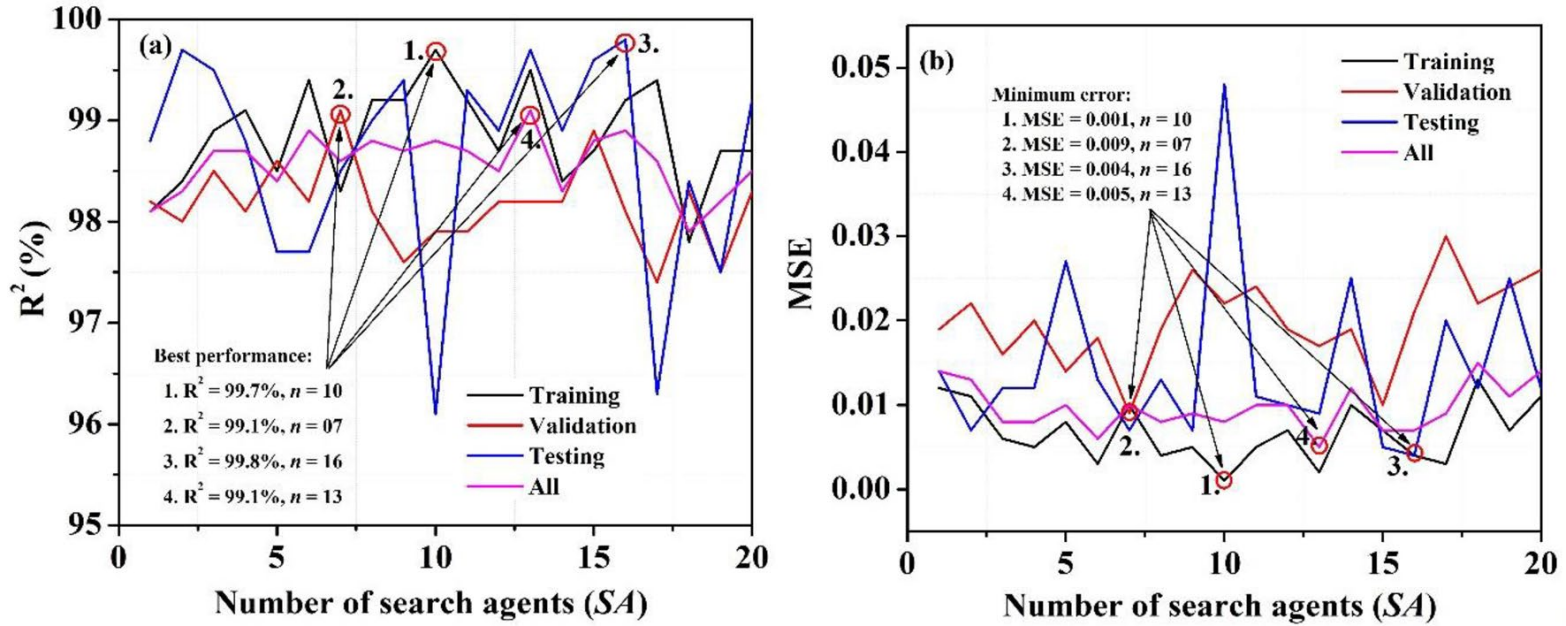
Illustration of variation in (**a**) regression coefficients (R^2^) and (**b**) MSE as a function of the number of search agents (*SA*) to explore the optimum value.

The second essential modeling parameter is the number of whale population/search agents (SA) that may play an important role in designing a perfect model. In order to do this, we varied the search agents one by one (*SA* = 1 to 20) and obtained a variety of results, which are shown in Table 3. For easier comprehension and evaluation, the results are also shown graphically in Fig. 6. We have observed that (*SA* = 10, 7, and 16) individually demonstrate the best performance for training, validation, and testing cases, respectively, while (*SA* = 13) yielded the best performance for all datasets. Finally, we have screened six models (with *SA* = 6, 8, 10, 13, 15, and 16), which achieved our selection criteria for best simulation results considering all datasets and recorded them.

**Table 3:** Permeate flux prediction at various stages using the proposed MWOA-ANN model.

Number of search agents (*SA*)	Results {*P*_flux_ (L/h m^2^)}							
	Training		Validation		Testing		All	
	R^2^ (%)	MSE	R^2^ (%)	MSE	R^2^ (%)	MSE	R^2^ (%)	MSE
1	98.1	0.012	98.2	0.019	98.8	0.014	98.1	0.014
2	98.4	0.011	98.0	0.022	99.7	0.007	98.3	0.013
3	98.9	0.006	98.5	0.016	99.5	0.012	98.7	0.008
4	99.1	0.005	98.1	0.020	98.8	0.012	98.7	0.008
5	98.5	0.008	98.6	0.014	97.7	0.027	98.4	0.010
6*	99.4	0.003	98.2	0.018	97.7	0.013	98.9	0.006
7	98.3	0.010	99.1	0.009	98.5	0.007	98.6	0.010
8*	99.2	0.004	98.1	0.019	99.0	0.013	98.8	0.008
9	99.2	0.005	97.6	0.026	99.4	0.007	98.7	0.009
10*	99.7	0.001	97.9	0.022	96.1	0.048	98.8	0.008
11	99.2	0.005	97.9	0.024	99.3	0.011	98.7	0.010
12	98.7	0.007	98.2	0.019	98.9	0.010	98.5	0.010
13*	99.5	0.002	98.2	0.017	99.7	0.009	99.1	0.005
14	98.4	0.010	98.2	0.019	98.9	0.025	98.3	0.012
15*	98.7	0.007	98.9	0.010	99.6	0.005	98.8	0.007
16*	99.2	0.004	98.1	0.021	99.8	0.004	98.9	0.007
17	99.4	0.003	97.4	0.030	96.3	0.020	98.6	0.009
18	97.8	0.013	98.3	0.022	98.4	0.012	97.9	0.015
19	98.7	0.007	97.5	0.024	97.5	0.025	98.2	0.011
20	98.7	0.011	98.3	0.026	99.2	0.012	98.5	0.014

The number of search agents (SA) was selected as the optimization modeling parameter.

*Significant predicted values for all datasets.

#### Dataset division optimization

We used dataset division (75 percent training, 20 percent validation, and 05 percent testing) as part of earlier recommendations by Gil et al.^35^ in the first and second optimizations to validate the proposed models. We found eight models that outperformed the Gil et al. (2018) model results (ANN and RSM). We have divided datasets into several combinations and recorded numerous useful outcomes, as indicated in Table 4, for a more indepth analysis. Finally, we screened two models (with dataset division = 70–15–15 and 80–00–20), which achieved our selection criteria and recorded them.

**Table 4:** Permeate flux prediction at various stages using the proposed MWOA-ANN model.

Dataset division (%)	Results {*P*_flux_ (L/h m^2^)}							
	Training		Validation		Testing		All	
	R^2^ (%)	MSE	R^2^ (%)	MSE	R^2^ (%)	MSE	R^2^ (%)	MSE
60–20–20	98.8	0.007	95.0	0.045	97.2	0.027	97.3	0.019
70–15–15*	99.9	⁓ 0.000	98.5	0.013	96.7	0.036	98.9	0.007
80–10–10	98.1	0.012	98.8	0.011	99.7	0.017	98.2	0.012
90–05–05	98.4	0.011	99.7	0.003	99.4	0.005	98.5	0.011
60–00–40	98.6	0.008	–	–	88.5	0.108	93.2	0.047
70–00–30	99.5	0.002	–	–	89.8	0.086	98.5	0.028
80–00–20*	99.9	⁓ 0.000	–	–	96.7	0.033	98.9	0.007
90–00–10	99.2	0.005	–	–	98.6	0.015	98.8	0.008

The optimization modeling parameter chosen is dataset division (%).

*Significant predicted values for all datasets.

### Best-optimized models

We developed ten models (MWOA-ANN Model-1 to Model-10) by regressive optimization (*n*, *SA*, and dataset division as variables), which are found to be superior to the existing RSM, ANN, and WOA-ANN models. As evident from Table 5, MWOA-ANN Model-6 has outperformed most with the least errors (0.005 L/h m^2^). We also noticed that all the ten considered models needed only one hidden layer, whereas Gil et al.^35^ models needed two to accomplish a reasonable extent of modeling efficiency. According to the literature, additional hidden layers complicate models. Therefore, our models are less complicated than the existing models. In summary, the modeling parameters (*n*, *SA*, and dataset division) assume critical significance in the modeling process and significantly impact the model’s success.

**Table 5:** Comparison of the modeling efficiency of the proposed models with that of the other models.

Desalination plant performance prediction {*P*_flux_ (L/h.m^2^)}									
Models	Optimization parameters							Results	
	Number of hidden layers (*H*)	Number of hidden layer nodes (*n*)	Activation function	Training function	Dataset division (%)	Error performance function	Search agents (*SA*)	R^2^ (%)	Error
[A] existing models									
RSM Model, Gil et al.^35^	–	–	–	–	–	–	–	98.5	0.100
ANN Model, Gil et al.^35^	2	{4:7:2:1}	*Logsig-logsig-purelin*	*Trainlm*	(75–20–05)	RMSE	–	98.8	0.060
[B] other models									
WOA-ANN Model-1	1	{4:10:1}	*Logsig-purelin*	*Trainlm*	(75–20–05)	MSE	15	98.8	0.008
WOA-ANN Model-2	1	{4:11:1}	*Logsig-purelin*	*Trainlm*	(75–20–05)	MSE	15	98.9	0.007
WOA-ANN Model-3	1	{4:13:1}	*Logsig-purelin*	*Trainlm*	(75–20–05)	MSE	15	98.9	0.007
[C] Proposed Models									
MWOA-ANN Model-1	1	{4:11:1}	*Logsig-purelin*	*Trainlm*	(75–20–05)	MSE	15	98.8	0.007
MWOA-ANN Model-2	1	{4:15:1}	*Logsig-purelin*	*Trainlm*	(75–20–05)	MSE	15	98.9	0.008
MWOA-ANN Model-3	1	{4:11:1}	*Logsig-purelin*	*Trainlm*	(75–20–05)	MSE	06	98.9	0.006
MWOA-ANN Model-4	1	{4:11:1}	*Logsig-purelin*	*Trainlm*	(75–20–05)	MSE	08	98.8	0.008
MWOA-ANN Model-5	1	{4:11:1}	*Logsig-purelin*	*Trainlm*	(75–20–05)	MSE	10	98.8	0.008
MWOA-ANN Model-6*	1	{4:11:1}	*Logsig-purelin*	*Trainlm*	(75–20–05)	MSE	13	99.1	0.005
MWOA-ANN Model-7	1	{4:11:1}	*Logsig-purelin*	*Trainlm*	(75–20–05)	MSE	15	98.8	0.007
MWOA-ANN Model-8	1	{4:11:1}	*Logsig-purelin*	*Trainlm*	(75–20–05)	MSE	16	98.9	0.007
MWOA-ANN Model-9	1	{4:11:1}	*Logsig-purelin*	*Trainlm*	(70–15–15)	MSE	13	98.9	0.007
MWOA-ANN Model-10	1	{4:11:1}	*Logsig-purelin*	*Trainlm*	(80–00–20)	MSE	13	98.9	0.007

*Best-of-best optimized proposed model.

### Best-of-best optimized model and their novelty

**Figure 7:**
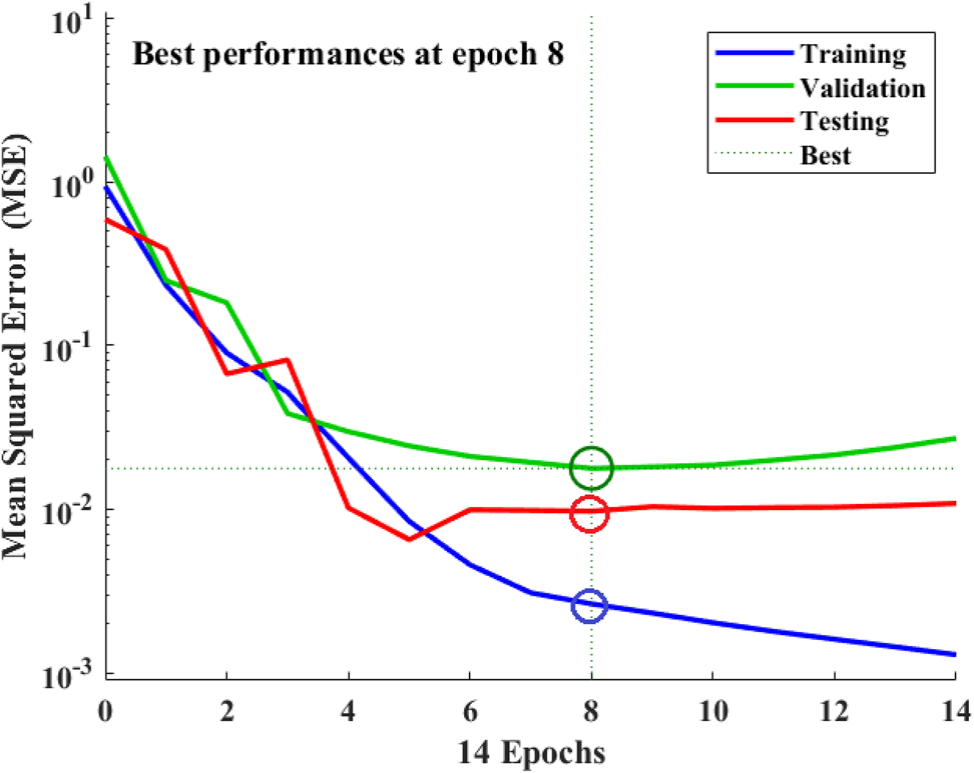
Best performances (training, validation, and testing) at epochs 8 of Model-6. *Used Neural Network Toolbox of MATLAB version 2019b for investigations.

**Figure 8:**
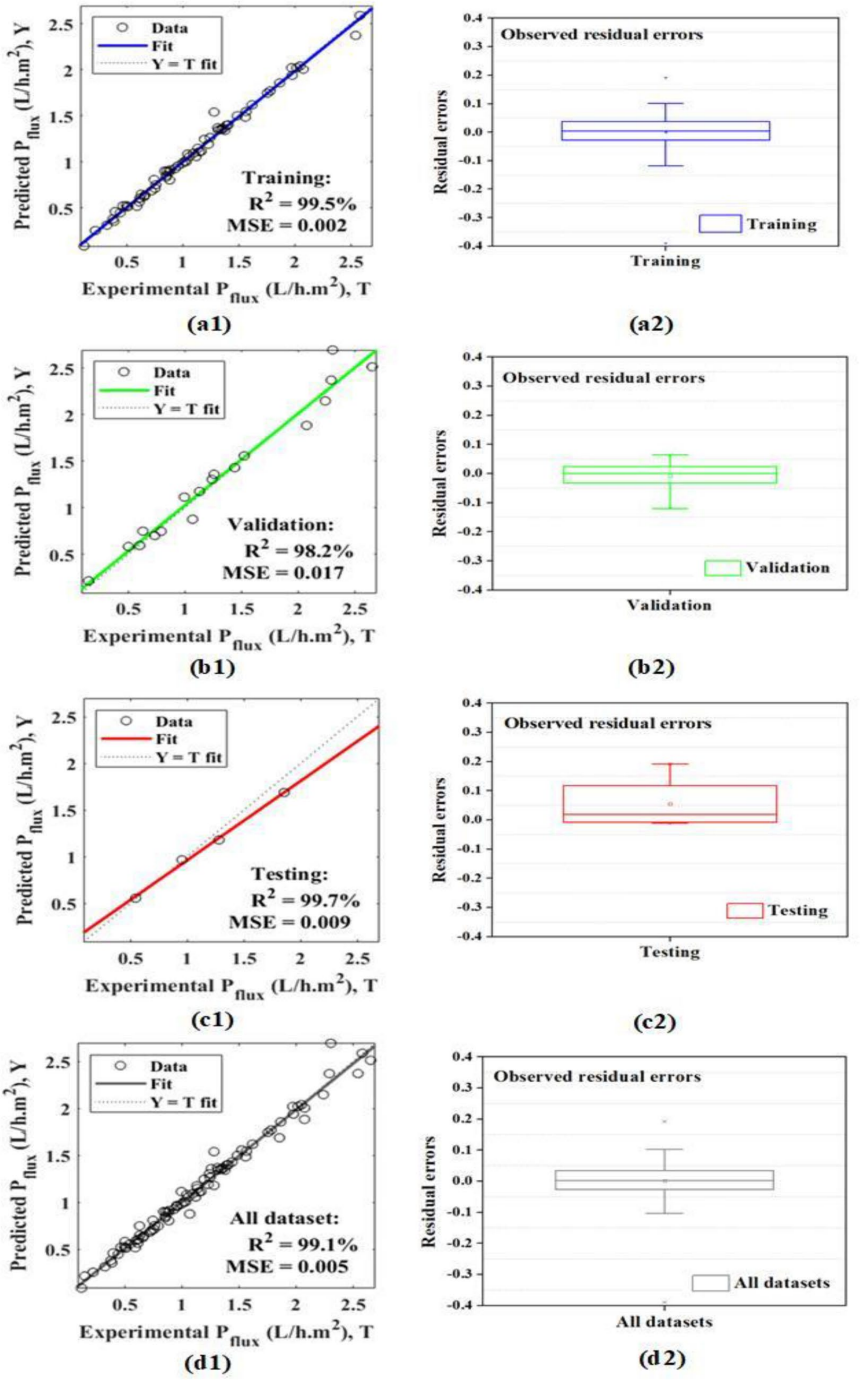
Scatter and observed residual errors box plots for Model-6: (**a1**, **a2**) training (**b1**, **b2**) validation (**c1**, **c2**) testing and (**d1**, **d2**) all datasets.

As shown in Table 5, the MWOA-ANN Model-6 outperforms the other ten proposed models, as well as the RSM and ANN models proposed in the literature for the same datasets. Hence, it is important to explore and express the novelty of this model in depth. All the proposed models have been developed in MATLAB version 2019b (Neural Network Toolbox). The simulation results reveal that this model shows the best performance (across training, validation, and testing stages) at epoch 8, as shown in Fig. 7. The performance results show fast convergence of the model. Further, the beauty of this model is that it displays excellent performance (R^2^ = 99.5%) with minimum error (MSE = 0.002) in the training stage, which is very close to zero, indicating a close fit with experimental as evident in Fig. 8a1. The residual errors observed in the training stage are quite reasonable and acceptable (span of − 0.1 to 0.1), as apparent from Fig. 8a2. Likewise, validation performance also recorded acceptable performance (R^2^ = 98.2%, MSE = 0.017) with residual errors (span of − 0.1 to 0.1), as illustrated in Fig. 8b1 and b2. Furthermore, an excellent testing performance is also noted (R^2^ = 99.7%, MSE = 0.009) with desirable residual errors (span of 0.0 to 0.2), as shown in Fig. 8c1 and c2. At last, all dataset performance also demonstrates acceptable outcomes (R^2^ = 99.1%, MSE = 0.005) with desirable residual errors (span of -0.1 to 0.2), as shown in Fig. 8d1 and d2. In summary, we conclude that Model-6 (R^2^ = 99.1%, MSE = 0.005, *H* = 1, *n* = 11, *SA* = 13) is most suitable for investigating the RO desalination plant’s performance with fast convergence and minimum error.

## Statistical evidence and validation of best-of-best optimized model 6

The performance of the RO desalination plant's experimental permeate flux was compared with the proposed model's predicted permeate flux to validate the best of the best-optimized Model 6. In this case, *t*-test was performed using 88 observations for both the experimental and predicted models. We noticed that the experimental permeate flux values match the predicted permeate flux estimates for the proposed Model 6. As a result, the proposed model was found to be valid by the experiment with a 96% level of significance (α = 0.05). As displayed in Table 6, the proposed models' *p*-values satisfy the *t*-test conditions (*p*-value < 0.05), have good Pearson correlation (0.99), and have desirably hypothesized mean differences of zero.

**Table 6:** Statistical validation using *t*-test of the proposed Model 6.

t-test parameters	Experimental permeate flux	Predicted permeate flux best-of-best optimized model 6
Mean	1.14	1.14
Variance	0.35	0.34
Observations	88	88
Pearson correlation	–	0.99
Hypothesized mean difference	–	0.00
*p*-value	–	0.01

## Conclusion

A hybrid Modified Whale Optimization Algorithm (MWOA) based Artificial Neural Network (ANN) models (MWOA-ANN) have been presented in this study. The humpback whale hunting behavior inspires the MWOA algorithm. It has three operators to simulate mathematically; search for prey, encircling prey, and bubble-net foraging. We employed it to explore the optimal weights and biases for ANN models, and the resulting hybrid models produced superior results than the non-hybrid ones (RSM, ANN) reported in the literature. The performance of the model for predicting permeate flux (L/h m^2^) of a reverse osmosis (RO) desalination plant was assessed in this study. There are 88 sets of input (4)—output (1) data collected from the literature. Ten models (MWOA-ANN Model-1 to Model-10) have been proposed to investigate the plant’s performance. According to simulation findings, all proposed models outperform existing ANN and response surface methodology (RSM) and hybrid WOA-ANN models. Among the ten proposed models, the MWOA-ANN Model-6 with a single hidden layer (*H* = 1), eleven hidden layer nodes (*n* = 11), and the thirteen search agents (*SA* = 13) produced the most outstanding regression results (R^2^ = 99.1%) with minimal errors (MSE = 0.005). The residual errors for Model-6 are also found to be within limits (span of − 0.1 to 0.2), further considering model efficiency. Finally, simulation findings demonstrate that the MWOA algorithm is an efficient optimizer that can outperform backpropagation (BP) and WOA algorithms in such cases of desalination plant modeling and may appear indispensable in similar process plants applications. During the simulations, possibility of limitations such as “Overfitting” are possible. However, it is effortlessly controlled by a step-by-step and systematic approach in this investigation. The MWOA-ANN hybrid model has been currently tested for 88 data sets provided by Gil et al.^35^. In the future, the authors shall conduct suitable RO-based desalination experiments to obtain higher number of datasets and explore the superiority of these hybrid models over previous models when considering huge data sets.

## Data Availability

The datasets generated during and/or analyzed during the current study are available from the corresponding author on reasonable request.
